# The Management of Anxiety and Depression in Pediatrics

**DOI:** 10.7759/cureus.30231

**Published:** 2022-10-12

**Authors:** Raena M Pettitt, Emma A Brown, Jordan C Delashmitt, Megan N Pizzo

**Affiliations:** 1 Department of Family Medicine, Liberty University College of Osteopathic Medicine, Lynchburg, USA

**Keywords:** adjunctive interventions, nonpharmacologic treatment, pharmacologic treatment, pediatric anxiety, pediatric depression

## Abstract

Anxiety and depression are among the most common psychiatric conditions affecting children and adolescents, and physicians in primary care settings often represent the first point of contact for these patients. Therefore, it is critical to provide these clinicians with an overview of current, evidence-based approaches for treating these conditions in pediatric and adolescent patients. Cognitive behavioral therapy (CBT) is an appropriate and effective first-line intervention for anxiety disorders in children and adolescents. For depressive disorders, treatment guidelines recommend either CBT or interpersonal therapy (IPT) as frontline treatment approaches. Pharmacologically, selective serotonin reuptake inhibitors (SSRIs) represent the most efficacious treatment for anxiety and depressive disorders in young persons. Combination therapies consisting of a psychotherapy plus an SSRI have produced greater therapeutic effects than either treatment alone. In particular, CBT plus sertraline is most effective in those with anxiety, whereas combining CBT or IPT with fluoxetine has been identified as the most effective treatment for depression in this population. Clinically, these combination therapies are especially useful in patients showing an insufficient response to treatment with only an SSRI or psychotherapy. A physician should also recommend lifestyle alterations to aid in the management of anxiety and depression, including diet, exercise, adequate sleep, limiting screen time, and spending time in nature. When used to complement standard treatment approaches, these interventions may provide the patient with additional symptom reduction while decreasing the return of symptoms in the long term.

## Introduction and background

Anxiety and depression are two common mental health diagnoses made in pediatric patients. The median age of onset for anxiety disorder is six years old and typically persists throughout the lifetime of those diagnosed. Generalized anxiety disorder (GAD) is characterized by excessive worrying, insomnia, irritability, fatigue, and decreased concentration. Other anxiety disorders include social anxiety disorder and separation anxiety disorder. The median age of onset for social anxiety disorder is 12 years old and is characterized by fear of embarrassment or scrutiny from peers. Separation anxiety disorder has an earlier onset at the age of eight years old and is characterized by extreme worry or anxiety when separated from someone with whom they have a firm attachment. Depression is usually diagnosed later in childhood, with a median age of onset of 13 years old [1,2]. Major depressive disorder (MDD) is characterized by low self-esteem, guilt, hopelessness, impulsivity, and changes in sleep and appetite. Children between the ages of three and five years old can have suicidal thoughts, but suicidal ideation has a higher occurrence in children 13 years old and older [3]. It is important for physicians to recognize these symptoms early so that optimal management of GAD and MDD can be provided and decreases the risk of developing other mood disorders [2].

## Review

Anxiety

Nonpharmacologic

Among the therapeutic approaches used in the treatment of this population, current evidence offers the most support to cognitive behavioral therapy (CBT), which is recommended as a first-line intervention due to its well-established efficacy and limited side effects [2,4-6]. While CBT remains the evidence-based current standard, other psychotherapies have also been effective in treating these patients, including social effectiveness therapy (SET) and acceptance and commitment therapy (ACT).

CBT is a combination of therapeutic approaches that teach the patient to identify feelings of anxiety and confront irrational thoughts during anxiety-provoking situations. Treatment with CBT focuses on cultivating mechanisms of modifying these thoughts and beliefs so as to alter maladaptive behaviors in response to distressing conditions [5,7,8]. Traditionally, CBT programs adopt a generic treatment approach that uses the same set of techniques to treat separation, generalized, and social anxiety disorders in children and adolescents [5,7,9,10]. However, this non-specific CBT approach is found to be less effective for young patients with social anxiety compared to other anxiety disorders [9-11]. Consequently, psychotherapies specifically targeting social anxiety disorders have been developed by altering existing therapies to address social skills deficits in this population [5,7,9,12].

Among the first of these tailored therapies was cognitive behavioral group therapy for adolescents (CBGT-A), a treatment approach that incorporates social skills training (SST) into group-based CBT programs [12,13]. Standard CBT programs are more effective in treating youth anxiety when SST is added [11,12]. In contrast, social effectiveness therapy for children (SET-C) is a group-based behavioral therapy for patients with social anxiety that focuses on exposure therapy, SST, and peer generalization [2,7,9,11-13].

ACT is a contemporary method that seeks to treat patients by promoting long-term, goal-oriented behaviors rather than focusing on symptom reduction [14,15]. Treatment with ACT focuses on developing psychological flexibility or the ability to accept experiences and commit to value-based behaviors [10,14-16]. ACT intervention has been proven to be effective in treating anxiety and depression with positive treatment outcomes comparable to those achieved by traditional CBT, which is the current standard for the treatment of anxiety disorders [9,10,14-16]. See Table 1 for a comparison of these psychosocial therapies.

**Table 1:** Psychotherapy interventions for anxiety disorders in children and adolescents *First-line treatment, particularly for mild to moderate cases [2,4-12,14-16]

Therapy	Indication	Most efficacious components	Predictors of greater treatment response
Cognitive behavioral therapy (CBT)*	Generalized, separation, and social anxiety disorders; ages ≥6 years old	Exposure therapy: the patient is repeatedly exposed to anxiety-provoking objects or situations, typically in a stepwise fashion of increasing intensity. Cognitive restructuring: challenging previously established thought patterns	Diagnoses of non-social anxiety disorders. Longer duration of overall treatment. Greater parental involvement when treating younger children. Positive expectations regarding the value of exposure-based CBT. The addition of social skills training
Social effectiveness therapy for children (SET-C)	Social anxiety disorder; ages 7-17	Exposure therapy: the patient is repeatedly exposed to anxiety-provoking objects or situations, typically in a stepwise fashion of increasing intensity. Social skills training: group-based learning of various social skills, including conversation, listening and telephone skills, and making and maintaining relationships	Decreases in child-reported loneliness throughout treatment
Acceptance and commitment therapy (ACT)	Generalized, separation, and social anxiety disorders; ages ≥6 years old	Psychological flexibility: the ability to accept experiences and commit to value-based behaviors. Exposure therapy: the patient is repeatedly exposed to anxiety-provoking objects or situations, typically in a stepwise fashion of increasing intensity. Acceptance: choosing to tolerate negative thoughts and feelings rather than avoiding or attempting to change them. Cognitive defusion: observing own thought processes	Higher pretreatment psychological flexibility

Pharmacologic

Compared to adults, the efficacy of psychopharmacotherapy has not been well studied in pediatrics. Currently, the only FDA-approved medication for the treatment of generalized anxiety disorder in children and adolescents is duloxetine, a serotonin-norepinephrine reuptake inhibitor (SNRI) [1]. Though duloxetine is approved by the FDA, studies have shown that selective serotonin reuptake inhibitors (SSRIs) are more efficacious than SNRIs and are considered the first-line pharmacologic treatment of GAD in pediatrics [17]. SSRIs, such as fluoxetine and sertraline, significantly improve GAD in children and adolescents and are well tolerated [1].

Benzodiazepines are a class of anxiolytics that have not been well investigated in pediatric patients. Studies indicate no significant difference between the drug and placebo in relief of GAD symptoms. In addition, adverse reactions such as irritability, drowsiness, oppositional behavior, dry mouth, and treatment-emergent suicidality were experienced [1,2]. Furthermore, children treated with benzodiazepines have an increased risk of upper and lower extremity fractures. A clear causation has not been identified, but it has been hypothesized that because benzodiazepines can cause dizziness, there is an increased risk of falling, which can lead to fractures [18].

Other anxiolytics traditionally used in adults have not been indicated for use in children due to harmful side effects or inefficacy. For example, tricyclic antidepressants (TCA) are not indicated, as potentially dangerous side-effects such as increased QT intervals and arrhythmias in those treated with this class of drugs have been observed [2]. Furthermore, buspirone has been observed to be well tolerated in children but does not show significant improvements in GAD when compared to a placebo [1,2].

A notable side effect of antidepressants is activation, which is described as a conglomerate of symptoms including impulsivity, restlessness, and insomnia. Children and adolescents at risk of bipolar depressive disorder and those diagnosed with attention-deficit/hyperactivity disorder (ADHD) are more likely to experience activation when being treated for anxiety with an SSRI. It has been recommended that pediatric patients at risk of activation should be prescribed a lower starting dose of SSRIs and then titrated slowly [19].

The protocol for discontinuing treatment with antidepressants is dependent on the half-life of the medication. For example, due to fluoxetine’s long half-life, the drug may be stopped without tapering. A small amount of fluoxetine will remain in the system for up to nine days after the patient stops actively taking the drug. Other medications, paroxetine and venlafaxine, with shorter half-lives may require tapering in order to avoid discontinuation syndrome, which is characterized by flu-like symptoms and mood changes [20].

Research investigating new pharmacologic agents’ efficacy in treating pediatric GAD shows promising results. Vortioxetine, an SSRI, significantly reduces GAD especially in patients also experiencing symptoms of depression. Vortioxetine has been observed to be well tolerated, but adverse effects can include headache, nausea, dysmenorrhea, and vomiting [1]. Furthermore, atomoxetine, a medication traditionally used to treat ADHD, has significantly reduced GAD in the pediatric population. Overall, atomoxetine has been well tolerated, but some children treated did experience increased heart rate, increased blood pressure, drowsiness, and decreased appetite. The changes in heart rate and blood pressure may require monitoring after each increase in dosing [2]. Guanfacine, which is approved to treat attention-deficit/hyperactivity disorder, has been noted to be effective in treating GAD in the pediatric population as well [21]. A sample list of medications indicated for the treatment of GAD in the pediatric population is summarized in Table 2.

**Table 2:** Pharmacologic treatment for children with generalized anxiety disorder *First-line treatment SSRI: selective serotonin reuptake inhibitor; bid: twice a day; SNRI: serotonin-norepinephrine reuptake inhibitor [2,22-26]

Medication	Drug class	Formulation	Dosing	Minimum duration	Potential side effects	Special notes
Fluoxetine*	SSRI	Capsule, tablet, or liquid	Starting dose: 10 mg/day; typical dose: 20-60 mg/day	4-8 weeks	Treatment-induced suicidality, increased BMI, and mania	Contraindicated with the use of tricyclic antidepressants, antiarrhythmic drugs, and neuroleptics. A longer half-life causes this drug to have the smallest occurrence of withdrawal symptoms
Sertraline*	SSRI	Capsule, tablet, or liquid	Starting dose: 25 mg/day; maximum dose: 200 mg/day	4-8 weeks	Headache, nausea, vomiting, abdominal pain, diarrhea, dyspepsia, and insomnia	Bid dosing is recommended in adolescents due to its short half-life. Decreased absorption with food. Well tolerated in children ages 6-17
Duloxetine	SNRI	Capsule or tablet	Starting dose: 30 mg/day; maximum dose: 120 mg/day	6-8 weeks	Weight loss, constipation, dry mouth, drowsiness, and increased heart rate	FDA approved

Depression

Nonpharmacologic

For the treatment of mild depression in young patients, treatment guidelines recommend a period of watchful waiting before beginning active intervention. If symptoms continue, evidence-based psychotherapy is the first-line of intervention for low-severity cases and in patients unable to take medication due to apprehension or contraindication. In contrast, antidepressants are often used to complement psychotherapies in treating more severe cases or patients in which first-line psychotherapy alone was ineffective [27-29]. The two psychological therapies most often used to treat childhood and adolescent depression are CBT and interpersonal therapy (IPT) [30]. In addition to these more established interventions, ACT is another modality that has been shown to be effective [14,15].

Better treatment outcomes have been observed in CBT programs that include any combination of cognitive restructuring, the involvement of caregivers, behavioral activation, and the increased participation in pleasurable activities [27-29]. Relatively less studied, IPT focuses on developing interpersonal problem-solving skills, modifying communication patterns, and improving relationships. As such, rather than attempting to reduce depressive symptoms directly, IPT seeks to address them by improving interpersonal relationships [27,30]. This therapeutic approach has been suggested to be particularly beneficial when there is a well-established relational component to the underlying cause of the patient’s depressive symptoms [29].

Both CBT and IPT are regarded as well-established treatments for MDD in adolescent patients over 13 years of age, though limited evidence exists for treatment in younger patients [27-30]. While more research has been devoted to CBT, IPT has been found to produce comparable effects [3,27,29]. ACT, as previously described, has also been successful in treating childhood and adolescent depression. When compared to CBT, treatment with ACT has been found to produce similar outcomes in this population [14,15].

Pharmacologic

SSRIs are the recommended first-line pharmacologic treatment for children with MDD. Fluoxetine has been observed to be efficacious in treating pediatric MDD and is well tolerated in children. Though adverse reactions are rare, the FDA recommends a follow-up visit four weeks following the initial treatment to evaluate effectiveness and treatment-induced suicidality [22]. Escitalopram and sertraline have also been found to be highly efficacious when treating children with MDD. Though not as well studied as fluoxetine, escitalopram has been recommended by the FDA as another first-line treatment for children with MDD. The efficacy of all SSRIs prescribed to pediatric patients should be evaluated between four and six weeks. If no change in symptoms is observed, it is recommended to evaluate dosing and treatment duration. Pediatric patients are often underdosed due to low body weight and may require a higher dose than expected [20,27].

SNRIs may also be prescribed to treat MDD in pediatric patients. Specifically, duloxetine may be efficacious in decreasing symptoms of depression in children, though the FDA indicates duloxetine for the treatment of pediatric GAD. Furthermore, it has been demonstrated that adverse side effects are more common with the treatment of SNRIs than with SSRIs [20,27].

Tricyclic antidepressants are efficacious in treating MDD in children, but adverse side effects render this class of drugs less favorable. Specifically, TCAs increase the risk of seizures in children and can cause QT prolongation and arrhythmias. If prescribed, children taking TCAs require periodic cardiac monitoring by electrocardiogram [20].

Potential adverse reactions to antidepressants must be considered before prescribing treatment. Similar to anxiety, children being treated for MDD with SSRIs are at risk of activation symptoms. For each one-year increase in age, there is a 27% decrease in the risk of activation. Family history of bipolar depressive disorder should be taken into consideration when prescribing SSRIs as this may increase the likelihood that the patient experiences activation. Furthermore, serotonin syndrome is a life-threatening reaction that may occur with the treatment of SSRIs. An increase in postsynaptic serotonin may result in symptoms including tachycardia, arrhythmias, hypertension, and diarrhea. The risk of serotonin syndrome increases when a patient is treated with multiple medications that affect serotonin levels in the synaptic cleft. Though rare, interactions between multiple serotonin-affecting medications should be reviewed to reduce the risk of serotonin syndrome [19,20]. A list of medications indicated for treating MDD in children is summarized in Table 3.

**Table 3:** Pharmacologic treatments for children with major depressive disorder *First-line treatment SSRI: selective serotonin reuptake inhibitor; TCAs: tricyclic antidepressants; SNRI: serotonin-norepinephrine reuptake inhibitor [20,27,30-32]

Medication	Drug class	Formulation	Dosing	Duration	Potential side effects	Special notes
Fluoxetine*	SSRI	Capsule, tablet, or liquid	Starting dose: 10-20 mg/day; typical dose: 20-80 mg/day	Initial treatment: 4-6 weeks; minimum duration for one depressive episode: 1 year	Treatment-induced suicidality, increased BMI, and mania	May be up to three times as potent in pediatrics than in adults. Approved for the treatment of ≥8 years old. Contraindicated with the use of TCAs, antiarrhythmic drugs, and neuroleptics. FDA recommends a four-week follow-up. FDA approved
Escitalopram*	SSRI	Capsule, tablet, or liquid	Starting dose: 5-10 mg/day; typical dose: 10-40 mg/day	Initial treatment: 4-6 weeks; minimum duration for one depressive episode: 1 year	Increased BMI and mania	Approved for the treatment of ≥12 years old. FDA approved
Duloxetine	SNRI	Capsule or tablet	Starting dose: 30 mg/day; typical dose: 40-60 mg/day	Initial treatment: 6-8 weeks	Weight loss, changes in systolic blood pressure, and changes in heart rate	First-line SNRI

The combination of pharmacology and psychotherapy interventions

Current treatment guidelines recommend CBT as the first-line treatment for anxiety disorders in children and adolescents experiencing mild symptoms. However, in more severe cases, the recommended treatment approach is CBT plus an SSRI, as numerous studies have indicated that this combination is the most effective intervention for anxiety in patients of ages 7-17 years old [6,7]. Specifically, CBT plus sertraline is more effective than interventions using only CBT or sertraline monotherapy. The advantage of this combined intervention was found in treating generalized, social, and separation anxiety disorders. Furthermore, the benefits of using this combination therapy were maintained at 24- and 36-week follow-up periods [2,7,9].

For the treatment of mild depression in young patients, treatment guidelines recommend a period of watchful waiting before beginning active intervention with either CBT or IPT. If symptoms persist, adding an SSRI to the patient’s treatment regimen may be required [27,29]. In moderate to severe cases of MDD, clinical practice guidelines recommend that the patient be treated with a combination of fluoxetine plus CBT or IPT [27,29,30]. Interventions that couple fluoxetine with psychotherapies have been found to produce the greatest therapeutic effects in young patients, with some evidence suggesting that the addition of CBT may help reduce the risk of treatment-induced suicidality [29,30].

Adjunctive interventions

In addition to psychotherapies and pharmacologic agents, several other interventions have been shown to improve symptoms of anxiety and depression in pediatric patients. These lifestyle interventions can benefit patients and are important to consider because depression and anxiety often persist throughout life after being diagnosed in children and adolescents. Diet and nutrition management, screen time and sleep recommendations, and nature-based interventions represent practical tools that a physician can aid patients in integrating into their daily lives.

Diet and Nutrition Interventions

Possibly the most easily integrated interventions that a primary care physician can advise in the management of anxiety and depression involve changes in dietary habits. Following dietary recommendations, avoiding certain products, and taking nutritional supplements all have the potential to improve patients’ symptoms. One study showed that patients consuming sufficient fruits, vegetables, grains, and milk products had significantly fewer visits to their primary care doctor to address mental health symptoms. Dietary recommendations for managing pediatric anxiety and depression are listed in Table 4. Compliance with each additional recommendation reduced the relative risk for mental health visits by 15% [33].

**Table 4:** Dietary recommendations for the management of pediatric anxiety and depression [33]

Food	Recommended amount
Fruits and vegetables	≥6 servings per day
Grain products	≥6 servings per day
Milk and milk alternatives	≥3 servings per day
Meat and meat alternatives	≥2 servings per day
Saturated fats	<10% of daily caloric intake
Added sugars	<10% of daily caloric intake

Adolescent symptoms of anxiety and depression can also be improved by completely stopping the intake of caffeine and cannabis products. There is a significant correlation between caffeine ingestion and anxiety and depression in children [34]. The avoidance of all cannabis products is also recommended in pediatric patients with anxiety and depression. While studies are still being done to evaluate the clinical impacts of cannabis, sufficient evidence demonstrates higher rates of anxiety and depression with its use in children. Because it has been shown that on average, one out of three children admits to having tried cannabis products, it is worthwhile for physicians to address the avoidance of these products during a visit [35].

In managing anxiety and depression, dietary supplements that can be considered adjunctive therapies include omega-3 fatty acids, probiotics, vitamin D, and folate. Supplementation with polyunsaturated fatty acids, such as omega-3 fatty acids, can help to correct dysfunction in the transport of neurotransmitters within the neuron. Because this dysfunction is associated with depressive symptoms, some studies have shown that supplementation with docosahexaenoic acid (DHA) and eicosapentaenoic acid (EHA) helps to improve and prevent symptoms of depression [36,37]. DHA and EHA supplementation of 1,000-2,000 mg per day for 12-16 weeks is recommended as an adjunctive therapy in the treatment of depression [38]. As studies continue to assess the impact of the gut microbiome on mental health, probiotics may also serve as a dietary supplement that is beneficial as an adjunctive therapy in the treatment of anxiety and depression in children. Studies have shown that a healthier gut microbiome is associated with decreased levels of inflammation and a healthier BMI. Because inflammation and a higher BMI are both associated with symptoms of depression, probiotics and a healthy gut microbiome can decrease symptoms of depression and improve the response to antidepressants [36]. Deficiency in vitamin D is also associated with depression, and treating this deficiency with supplementation has also been shown to improve symptoms [36]. Lastly, folate supplementation has been shown to improve depression symptoms in adolescents, specifically when there is a mutation in the methylenetetrahydrofolate reductase gene [37].

Exercise Interventions

Exercise interventions are among the most studied adjunctive therapies and have proven very effective in reducing symptoms of anxiety and depression in pediatric populations. Furthermore, studies have shown a significantly higher adherence to exercise treatment interventions in adolescents as compared to psychological and drug therapies [39]. Yoga is particularly effective in the reduction of symptoms of anxiety [39,40]. For the most significant improvement, one study recommended participating in yoga sessions at least four times a week for at least six weeks [40]. Similarly, aerobic exercise has been repeatedly shown as an effective treatment to reduce depression and depressive symptoms [39,41-44]. Current guidelines recommend moderate to vigorous intensity exercise for 60 minutes or more per day for children and adolescents. Studies have shown that patients meeting or exceeding this guideline demonstrate the greatest reduction in depressive symptoms [43,45,46]. Participating in sports is one way to make this goal more feasible and has also been shown specifically to reduce symptoms of anxiety and depression as well [45].

Screen Time Limitation and Sleep Recommendations

Studies have shown a correlation between increased screen time and the increased severity of anxiety symptoms and the duration of depressive symptoms [47,48]. Specifically, for maximal benefit, it is recommended that, when possible, screen time is limited to less than two hours a day [47]. One study found that adhering to this screen time limit and getting between nine and 11 hours of sleep significantly reduced the number of doctor visits addressing mental health concerns [33].

Nature-Based Interventions

As a consequence of spending more time with technology, children are spending less time in natural environments, which may contribute to the increased prevalence of anxiety and depression in pediatric populations. Both anecdotal evidence and clinical studies have found exposure to nature to be significantly beneficial to overall mental health. It is estimated that protecting natural areas and outdoor environments saves an average of six trillion dollars globally on healthcare costs associated with poor mental health [49]. Additionally, studies have shown that the benefits of nature-based activities are significantly greater than the benefits of exercise alone [50]. This information has led to a movement of greater incorporation of a variety of different types of outdoor therapies and nature-based interventions. In general, any increase in accessibility, exposure, and engagement with natural outdoor spaces for kids significantly improves their mental health [51]. These positive impacts of nature-based interventions are even greater for kids that are of lower socioeconomic status and those that live in areas with a higher population density [52,53]. The specific intervention can vary based on a patient’s needs, ranging from visiting local parks with greater frequency to outdoor programs, camp experiences, and wilderness expeditions. For some patients, it may be beneficial to recommend a structured wilderness therapy program facilitated by a trained professional. These types of programs offer group treatment and are uniquely beneficial due to the incorporation of natural environments [51].

Osteopathic Manipulative Interventions

While there are currently no studies evaluating osteopathic manipulative treatments for anxiety and depression in children and adolescents, the following techniques are known to improve heart rate variability by downregulating the sympathetic nervous system and could prove beneficial in reducing symptoms of anxiety and depression: cervical soft tissue/long axis kneading, cervical high velocity/low amplitude, sacral decompression, suboccipital/occipitoatlantal (OA) decompression, doming the respiratory diaphragm, and the compression of the fourth ventricle [54].

## Conclusions

As the prevalence of anxiety and depression increases in the pediatric population, there are a variety of therapies and interventions that a primary care doctor may utilize in managing these conditions. For many patients, the use of pharmacologic agents, referral to a mental health professional for psychosocial therapy, adjunctive interventions, and coaching regarding lifestyle modifications collectively represent the most effective management. It is important for the treating physician to refer to mental health professionals for the administration of psychosocial therapies when needed. This type of referral may be necessary during the continual evaluation of the efficacy of managing a child’s anxiety or depression.

While there are a variety of therapies that are available to treat these conditions, there are still relatively few studies looking at pharmacotherapy in the pediatric population as compared to adults. In addition, the management of these conditions could be improved by studies evaluating the most effective components of CBT that offer the greatest therapeutic response. These types of studies could be helpful in minimizing the use of pharmacologic agents, thereby reducing the risk of adverse reactions.
